# The Role of Hydrogen Bonding in the Folding/Unfolding Process of Hydrated Lysozyme: A Review of Recent NMR and FTIR Results

**DOI:** 10.3390/ijms19123825

**Published:** 2018-11-30

**Authors:** Domenico Mallamace, Enza Fazio, Francesco Mallamace, Carmelo Corsaro

**Affiliations:** 1 Dipartimento di Scienze Matematiche e Informatiche, Scienze Fisiche e Scienze della Terra (MIFT), Università di Messina, 98166 Messina, Italy; mallamaced@unime.it (D.M.); enfazio@unime.it (E.F.); ccorsaro@unime.it (C.C.); 2 Department of Nuclear Science and Engineering, Massachusetts Institute of Technology (MIT), Cambridge, MA 02139, USA; 3 Istituto dei Sistemi Complessi (ISC)—CNR, 00185 Rome, Italy

**Keywords:** protein denaturation, FTIR, NMR, hydration water, hydrogen bonding, energy landscape

## Abstract

The biological activity of proteins depends on their three-dimensional structure, known as the native state. The main force driving the correct folding mechanism is the hydrophobic effect and when this folding kinetics is altered, aggregation phenomena intervene causing the occurrence of illnesses such as Alzheimer and Parkinson’s diseases. The other important effect is performed by water molecules and by their ability to form a complex network of hydrogen bonds whose dynamics influence the mobility of protein amino acids. In this work, we review the recent results obtained by means of spectroscopic techniques, such as Fourier Transform Infrared (FTIR) and Nuclear Magnetic Resonance (NMR) spectroscopies, on hydrated lysozyme. In particular, we explore the Energy Landscape from the thermal region of configurational stability up to that of the irreversible denaturation. The importance of the coupling between the solute and the solvent will be highlighted as well as the different behaviors of hydrophilic and hydrophobic moieties of protein amino acid residues.

## 1. Introduction

**Figure 1:**
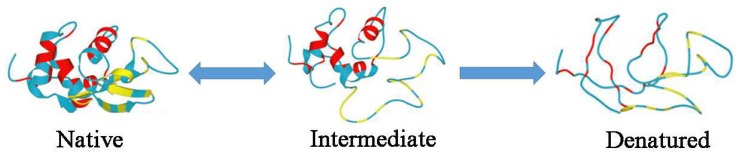
Schematic representation of the three different states of the protein lysozyme. The protein native state can be recovered from the intermediate state, which however, being unstable, can end in the irreversibly denatured state. Figure adapted from Reference [16,17].

Proteins are essential for life because they are able to perform and manage biological functions in such a complex way that much care is needed for the comprehension of these mechanisms at different levels. Although each protein accomplishes a specific task, we also need to be able to describe their common properties and characteristics. Proteins, consisting of complex linear chains of amino acid residues, can be considered as polypeptides. The amino acid residues, depending on their position in the chain, interact with each other and can form collapsed structures such as α-helices or β-strands. Indeed, proteins are characterized by a stable three-dimensional structure, called the native state, indicating the precise way of folding of that protein, and ultimately, the biological activity developed by the protein itself [1,2]. Although a protein with 100 residues needs from milliseconds to seconds in order to fold, the number of possible configurations is in the order of 10${}^{70}$, which corresponds to a folding time in the order of about 10${}^{52}$ years [3]. This inconsistency, known as Levinthal’s paradox [4], was, and actually is, the subject of numerous studies that try to address this important issue [5,6]. The main consideration concerns the existence of different interactions between amino acids with a different weight in term of strength and priority. These selective interactions, together with random thermal motions, induce fast conformational changes with a progressively lower potential energy that allow the formation of the native structure of proteins. Indeed, not every configuration will be explored but only those that lead to the most stable configuration. This mechanism is well illustrated by a funnel-shaped energy landscape [1,7,8,9]. When proteins are heated beyond the so-called denaturation threshold, they unfold irreversibly. Above the irreversible denaturation threshold, proteins lose their three-dimensional structure becoming a linear chain of amino acids and do not function any more. Furthermore, denatured proteins manifest heavy configurational changes, loss of solubility and aggregation processes. The unfolding process is strongly dependent on the temperature and on the time that proteins remain (permanence time) at that temperature. Under certain thermodynamic conditions (e.g., pressure and concentration), it can be reversed allowing proteins to re-fold in the right way [10,11,12]. Hence, upon heating above a certain temperature in the range 40–60 ${}^{{}^{\circ}}$C, the protein native state undergoes rapid conformational changes before it unfolds completely. These structural changes characterize the so-called intermediate state (see Figure 1) populated by a broad range of structures resulting from competing mechanisms, such as hydrophilic and hydrophobic interactions [13], and large-scale motion [14]. This competition depends on the temperature, and recently the value of about 280 K has been identified as the onset temperature of the hydrophobic effect [15].

The intermediate state is highly unstable [18] and sometimes the folding pathway of proteins is altered: proteins do not fold correctly and assume a *wrong* three-dimensional structure (misfolding). Actually, there is much interest in determining the cause of misfolding because it is one of the origins of many degenerative illnesses, such as Parkinson’s and Alzheimer’s diseases [19]. Comprehension at a microscopic level of the different mechanisms involved in the folding/unfolding process has indeed a primary role in understanding the causes and eventually finding the remedy to stop or slow down the advance of the wrong reactions/interactions. Finally, the so-called protein folding problem concerns the understanding of the physical processes that drive the polypeptide chain towards the lowest free energy state [8,20] which is determined by the balance between enthalpic and entropic costs involving both the protein and its hydrating solvent. Water, thanks to its abilities in developing hydrogen bond networks, is essentially the unique solvent able to control the structure, stability, dynamics, and thus the function of biomolecules. More profoundly, water molecules interacting with a protein may have both a structural and a dynamical character. Water molecules that are located within the protein internal cavities constitute part of the protein structure. Moreover, water molecules that cover the protein surface and make part of the so-called first hydration layer, allow protein residues to have the right dynamical flexibility that determine its specific biological activity [21]. In this context, the study of the properties and the influence of hydration water with respect to the proteins structure and dynamics, is ultimately fundamental to having a clear picture of the mechanisms involved in the folding/unfolding processes.

Since the water monolayer covering the protein surface is essentially a bidimensional hydrogen-bonded network connecting the different water clusters and the hydrophilic moieties of the protein surface [22,23], the protein dynamics, and thus its function, is slave to water dynamics [24,25,26]. Hence, the magnitude of the self-diffusion of hydration water strictly influences the large-amplitude motion of protein residues that is needed for the corresponding functionality [27,28].

Actually, the comprehension of the various kinds of interactions occurring between the different protein moieties and the solvent is an intriguing open question [29]. It is fundamental to analyze the interplay between hydrophilic and hydrophobic interactions that moves the thermodynamic properties of the system toward the corresponding equilibrium state. This interplay is strongly influenced by the temperature favoring the formation of hydrogen-bonded structures on cooling. Note that the secondary structure of proteins such as α-helices and β-sheets builds up by means of hydrophilic interactions and indeed hydrogen bonds [30], except for various membrane proteins for which the additional interaction with the lipid bilayer must be taken into account [31,32]. In order to focus such issues, proper experiments must be planned. It is well accepted that internal water favors the first steps of protein folding but at the end is partially thrown out from the inner core through cooperative hydrophobic interactions mediated by the corresponding hydrophobic moieties of proteins [33,34].

When water solvates amphiphilic molecules, the interplay between hydrophilic and hydrophobic interactions provokes a decrease in entropy with the subsequent build-up of well defined geometric structures such as ellipsoids, cylinders, layers, bilayers, etc. [35]. Thermodynamical variables, chemical composition, and the high directionality of hydrogen bonds determine the final geometry and the three-dimensional structural arrangement [36,37,38].

Recent theoretical advances allowed the prediction of protein structure starting from the knowledge of the amino acid sequence, thanks to the inclusion of water-mediated interactions for the study of the folding process [39]. One of the most promising approaches for the interpretation of the protein folding problem is based on a statistical approach that describes the protein’s potential surface as a rugged funnel-like landscape [40]. This latter approach is known as the free energy landscape theory, and by means of the same statistical approach used for the description of disordered systems, polymers, and phase transitions of finite systems [41,42,43,44], it considers that the folding process proceeds towards the formation of an ensemble of well defined structures finally “precipitating” in the protein native state [40].

**Figure 2:**
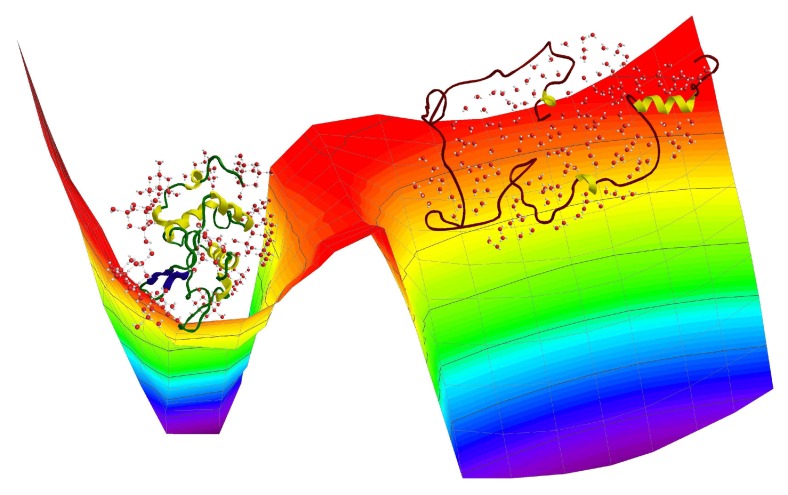
Three-dimensional simplified representation of the energy landscape for hydrated lysozyme, with only two wells of different height and width. At lower temperature (left well), the protein is in its native state and water molecules can develop a soft tetrahedral network surrounding the protein surface. At higher temperature (right well), the protein is an almost linear chain of amino acids and water molecules are essentially free.

Figure 2 reports a schematic energy landscape with only two wells with different heights and widths corresponding to the high-temperature conformation of an almost linear chain of amino acids (right side) and to the stable native configuration with the corresponding three-dimensional structure (left side) [45,46]. It is worth noting that our results, discussed in the next section, show that hydrogen bonds are effective and “incisive" only for the lowest well and here water molecules can develop a soft tetrahedral network surrounding the protein surface. On the contrary, at high temperature, all water molecules are essentially free and hydrogen bonds are not able to form between either protein residues or water molecules.

Herein, we review our recent results obtained by means of Nuclear Magnetic Resonance (NMR) and Fourier Transform InfraRed (FTIR) spectroscopies concerning the investigation of the mechanisms that intervene in and determine the evolution of the folding/unfolding process of hydrated lysozyme. Lysozyme is a small globular protein of about 15 kDa with antibacterial properties and was discovered by Fleming in 1922 during a search for medical antibiotics. Being ubiquitous among living organisms, lysozyme is probably the most studied enzyme in many different scientific fields including physics, biology, and medicine both theoretically and experimentally [11,12,16,18,19,22,26,47,48,49,50,51,52,53,54].

We make use of the mentioned experimental probes in order to obtain new microscopic insights for a better elucidation of those processes and interactions that determine the evolution of the protein folding/unfolding process. In fact, even when working on two different energy/time scales, both techniques provide microscopic details for water and/or the individual functional groups. We report our findings obtained by exploring the Energy Landscape from the thermal region of configurational stability, i.e., that of the protein native state, up to that of the irreversible denaturation where the protein reverts back to its unfolded state, a linear chain of amino acids. By means of different kinds of experimental investigations we figure out the role of water in mediating the relevant interactions as well as the importance of hydrophobic moieties for a correct folding pathway.

Overall, the aim of this review is to bring together the knowledge acquired to give a deeper comprehension of the investigated processes. This is done by considering existing interpretations and by suggesting future perspectives.

## 2. Results and Discussion

As a result of the seminal work by Rupley and Careri [55], today it is well accepted that at least one water monolayer surrounding the protein surface is essential in order for proteins to be active enough. The hydration level (h = grams of water/grams of dry protein) corresponding to this condition is around 0.3, allowing a complete development of the complex network of hydrogen bonds surrounding the protein surface that is able to endow the proper dynamics to the amino acid residues. In fact, although it has been shown that the enzymatic activity can exist even at very low-hydration levels [56], it increases dramatically for h ≥0.3, when all protein units have their own hydration shell [57,58].

Both structural and dynamical investigations reveal that water molecules have a crucial role in determining the thermal limits of activity for biomolecules. Its properties and tasks render water the 21st amino acid and its interactions with hydrophilic and hydrophobic peptide groups are the key to understanding the microscopic mechanisms of the protein’s folding/unfolding process. In the following subsections, how the physical properties of water trigger the protein function will be analyzed. Thereafter, the different interactions leading to different behaviors for protein hydrophilic and hydrophobic moieties will be pointed out.

### 2.1. Specific Heat and Self-Diffusion of Hydrated Lysozyme

**Figure 3:**
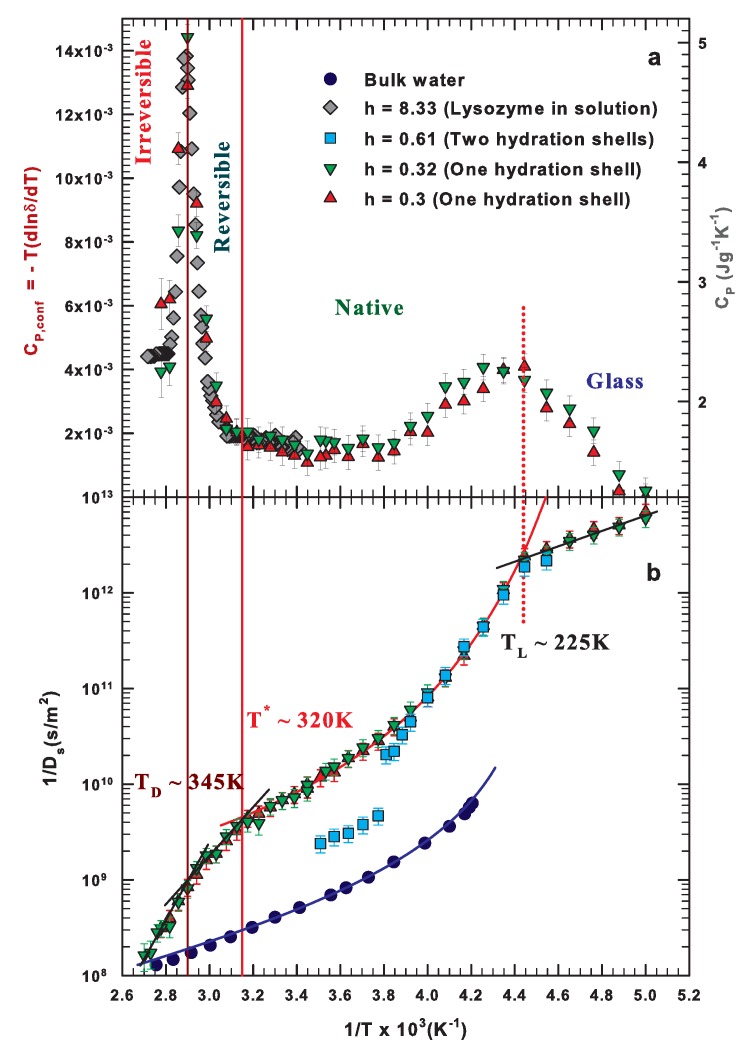
(**a**) The isobaric specific heat measured by means of temperature modulated scanning calorimetry for lysozyme in solution with h = 8.33 (grey diamonds; right axis) [10], and the configurational specific heat evaluated with static Nuclear Magnetic Resonance (NMR) by means of the water proton chemical shift for lysozyme with a single hydration shell, h = 0.32 and 0.3 (green triangles down and red triangles up, respectively; left axis) [59,61]. (**b**) The reciprocal of the self-diffusion coefficient for bulk water (blue circles) and lysozyme hydration water with h = 0.61 (cyan squares), 0.32 (green triangles down) and 0.3 (red triangles up) measured by means of the pulsed field gradient NMR technique [60]. The panels share the same *x*-axis, i.e., the inverse temperature. Figure adapted from Reference [59,60].

In this section, we report the calorimetric and self-diffusion investigations (Figure 3a,b, respectively) on hydrated lysozyme in a wide temperature range from the deep supercooling regime (T ≈ 200 K) to above the irreversible denaturation threshold (T ≈ 360 K) [59,60].

Both panels unambiguously evidence the existence of three characteristic temperatures for this system that correspond to important changes both from structural and dynamical points of view. The specific heat shows two maxima at about 225 K and 345 K and the onset of an additional energetic contribution at about 320 K. The existence of these two maxima is correlated with strong configurational changes occurring in the system at the corresponding temperatures. It is worth mentioning that the reported specific heat (Figure 3a) compares well with the data from two different experimental techniques and for systems with different hydration. On the other hand, the self-diffusion coefficient shows three corresponding changes in the dynamics at the same temperatures. Furthermore, in Figure 3b, there is a comparison among different data and in particular bulk water and protein hydration water at different hydrations. The two quantities are linked within the Adam–Gibbs theory that relates the relaxation time to the configurational entropy [59,62,63]. Indeed there is a relation between the self-diffusion coefficient and the configurational specific heat that implies the occurrence of corresponding changes in their behavior at the same temperatures. On the basis of this approach, for what concerns the thermal limit of biological activity, the low-temperature threshold, T${}_{L}≃225$ K, corresponds to what was originally named the protein glass transition, below which the hydrogen-bonded network becomes too rigid to allow protein motions and the whole system can be considered in a glassy state. It corresponds to the temperature of the so-called water dynamical crossover at which point the population of local low-density water structures dominates over that of high-density local structures [27,35,52,53,60,64,65]. This change in the water density and in the corresponding dynamics of the hydrogen-bonded network triggers the amino acids motion and the protein activity. Water plays the role of plasticizer of displacements from which the functional processes depend [66]. This dynamical transition was observed in a wide number of aqueous biosystems, including DNA, by means of different experimental techniques [26,35,53,64,67,68,69,70,71]. Although some experimental results have been questioned invoking instrumental resolution problems [53,65,72,73,74], the observation of an enhanced dynamics for temperatures higher than about 225 K is associated with the onset of protein biological activity triggered by the water dynamical transition. In fact, dry proteins (or those with a hydration lower than about h = 0.2) do not show any enhanced dynamics.

The ability to form well organized structures as temperatures decrease and the existence of different ensembles of structures make water a polymorphic system in all of its phases [75]. In particular, the liquid phase can be considered a mixture of high- and low-density structures whose relative population depends on the thermodynamical conditions. Thus, this mentioned dynamic crossover corresponds to a transition from a high-density liquid (high T), to a low-density liquid (arranged in clusters and/or network) at low T. This transition is known as the Liquid-Liquid Phase Transition of water and is related to its hypothesized second critical point [76,77,78].

On the contrary, the high-temperature threshold, T${}_{D}≃345$ K, essentially defines the limit of irreversible denaturation. Above T${}_{D}$, the hydrogen bond lifetime is so short that it does not allow the formation of water clusters and bridges between protein residues, provoking a complete unfolding of the protein. Water molecules become essentially free and their dynamics resemble that of bulk water as testified by the value of the self-diffusion coefficient at the highest temperatures (Figure 3b). T${}_{D}$ corresponds to another dynamic crossover of hydration water as evidenced by the self-diffusion data and observed also by neutron scattering and Molecular Dynamics (MD) simulations [54,79]. Note that both experimental results evidence important changes at another relevant temperature that is T* ≃320 K corresponding to the onset of tetrabonded water structures [80]. Above this temperature, the hydrogen bond lifetime is too small (less than picoseconds) and water behaves as a simple liquid. In fact, the thermal behavior of self-diffusion can be described by an Arrhenius law only for T < 225 K and for T > 320 K (also for bulk water) whereas between these two temperatures it follows a super-Arrhenius behavior very similar to that of bulk water, well fitted by a power-law with an exponent approximately equal to 2 [59,60,81]. Furthermore, additional and important information is obtained by looking at the diffusion data for h = 0.61. This value corresponds essentially to two hydration shells for lysozyme, below about 260 K a dramatic slow down occurs. It is due to the freezing of the second hydration shell, while the first remains liquid, as testified by the data that superimpose to those of h = 0.32 and 0.3 on subsequent cooling. This experimental observation assigns a bio-protective role to the first hydration shell and confirms the needs of at least a water monolayer in order to activate the protein biological functions [60]. The temperature of about 320 K coincides with the temperature at which the isothermal compressibility of water shows a minimum (that does not depend on the pressure) and with the crossing temperature of the coefficient of thermal expansion values measured at different pressures [80].

In summary, it is a thermodynamically consistent temperature that marks the onset of the water hydrogen bond clustering and of the corresponding network whose thermal development is described by super-Arrhenius behavior. In fact, for temperatures lower than T*, the water dynamics (e.g., the self-diffusion coefficient) is well described by means of a power law function in the frame of the Mode Coupling Theory (or of its extended version) [82,83]. This holds also for the protein hydration water. For temperatures higher than T*, water behaves almost like a simple liquid and has a dynamics with only one energy scale (the Arrhenius energy) as confirmed by the deviation of the bulk water data from the power law curve for T > T* in Figure 3b. The same happens for the protein hydration water but with an additional step at the denaturation threshold above which the internal bound water molecules also become free to move. In the next section we enter into the details of the role played by hydration water and hydrogen bonding in driving the reversibility of the lysozyme folding/unfolding process.

### 2.2. The Role of Water: NMR Results

We will show the results of NMR experiments on hydrated lysozyme with h = 0.3 by using the HR-MAS (High Resolution Magic Angle Spinning) technique by executing different thermal cycles of heating and cooling for the exploration of the whole folding/unfolding process [11,50,51]. One of the main findings of this investigation is that when lysozyme is in the native state, the water NMR signal is characterized by a single contribution, well represented by the canonical spectral transfer function of NMR, i.e., the Lorentzian function. Above a temperature of about 345 K and for the cooling after reaching this or higher temperatures, the water signal consists of two contributions belonging to internal (bound) and external (bulk-like) water [51]. This is consistent with the results obtained by looking at the corresponding FTIR spectra, illustrated in the following, in which the different local structures of water molecules can be identified [59,60,64].

**Figure 4:**
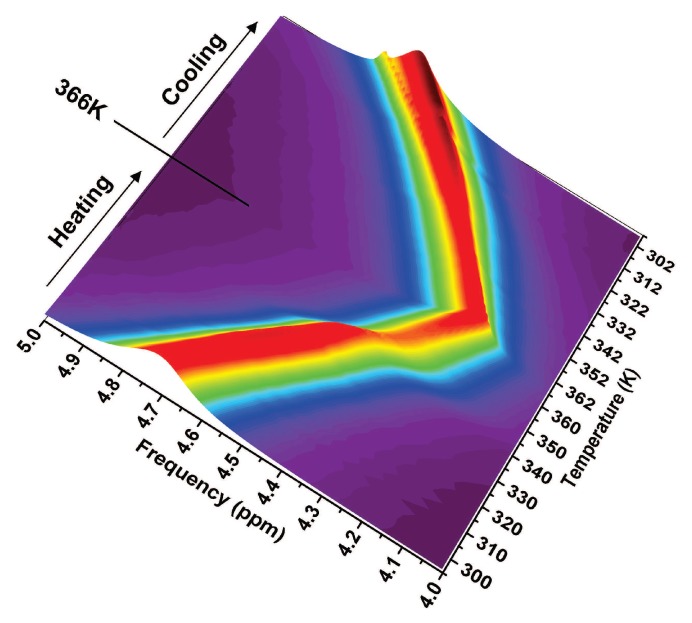
Three-dimensional color map of ${}^{1}$H HR-MAS spectra in the chemical shift region of the water NMR signal for hydrated lysozyme during the complete thermal cycle showing the irreversible denaturation: heating from 296 to 366 K and then cooling down to 298 K with 2 K steps. Note the sharp inflection at about 345 K only during the heating path.

In Figure 4, the HR-MAS spectra for the complete thermal cycle, heating from 296 to 366 K and then cooling down to 298 K with 2 K steps, are shown in a three-dimensional color map figure. A sharp variation at about 345 K is well evident during the heating, whereas the cooling shows continuous behavior. From the analysis of the spectral features such as peak position, width, and intensity, we were able to derive the role of water within the whole process and in its reversible character [51].

There is a marked hysteresis in all the observables if the cycle surpasses the temperature of 345 K, confirming the irreversibility of the process for T > T${}_{D}$. The thermal region of reversibility lies in the interval 320 < T < 345 K, where the system is in a metastable state and the native state could be recovered on cooling depending on the permanence time at the considered temperature. For what concerns the peak intensity (or magnetization) and the apparent spin-spin relaxation time (the inverse of the full width at half maximum), they show a similar trend with temperature with a maximum at about 325 K and a minimum at about 345 K, thus confirming the importance of these two temperatures in marking the thermal reversibility thresholds for the lysozyme folding/unfolding process [50,51]. In fact, variables belonging to thermal cycles executed up to 320 K show no hysteresis because the hydrogen bond lifetime is long enough to maintain stable structures and indeed the native state of the protein. On the contrary, above 320 K, the hydrogen bond lifetime decreases too much allowing a progressive increase in the amplitude of protein motion that finally ends in the loss of the structural conformation.

**Figure 5:**
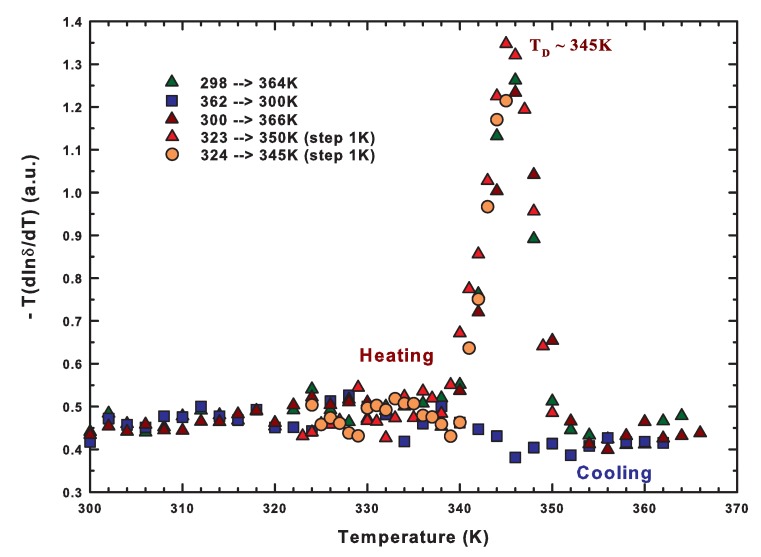
The configurational specific heat obtained by the derivative of the natural logarithm of the proton chemical shift (measured by ${}^{1}$H HR-MAS NMR) with respect to the temperature. We report both the heating and cooling paths for the complete cycle that goes beyond the denaturation and the heating path of other partial cycles. Figure adapted from Reference [51].

The peak position of each NMR signal, that is the chemical shift, is a very important quantity related to the structure of the studied system. In fact, it is well known that the shift with respect to the precessing Larmor frequency depends on the chemical environment experienced by each nucleus. For those systems that have important configurational degrees of freedom like water, if referred to an isolated molecule, it corresponds to the number of possible configurations. In such a case, the thermal derivative of its logarithm is proportional to the configurational specific heat as already shown in Figure 3a. Here, we show in Figure 5, the results for both the heating and cooling paths for the complete cycle that goes beyond the denaturation, and for the heating path of other partial cycles.

The steep inflection at about 345 K (shown in Figure 4) on heating causes the presence of a sharp maximum in the configurational specific heat, whereas the continuity of the behavior shown in the cooling path corresponds to a flat configurational specific heat. The presence of such maximum on cooling confirms the overall previous results shown in Figure 3a but points out a few differences with respect to the reported calorimetric results. The peak in the configurational specific heat that we have evaluated is narrower and more symmetric than that measured by temperature modulated scanning calorimetry [10]. This last quantity, different from the other, shows values for T > 350 K higher than those for T < 320 K. Furthermore, the cooling phase is nearly flat only in the case of the configurational specific heat. The cause of these discrepancies is that all the energetic degrees of freedom of the whole system are measured by calorimetric techniques, while only the configurational degrees of freedom of water molecules are taken into account by the proton NMR chemical shift. Hence, we are able to focus just on the microscopic configurational changes involved during the folding/unfolding process. On the other hand, the occurrence of the same specific heat maximum in both data confirms the relevance of structural changes induced by water in driving the lysozyme denaturation. We want to stress that, as reported in the Figure 5, when the maximum is overcome, no configurational changes occur during the cooling phase, indicating that the protein remains in the unfolded state as a linear chain of amino acids.

### 2.3. The Role of Water: FTIR Results

**Figure 6:**
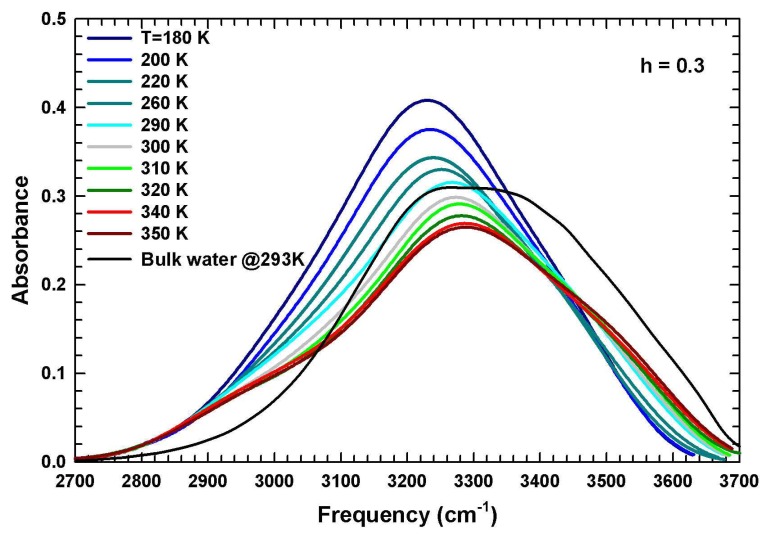
The O–H stretching vibrational band of hydrated lysozyme with h = 0.3 at several temperatures in the range 180 K < T < 350 K. The data of bulk water at 293 K (black line) are also reported for comparison. Figure adapted from Reference [84].

The microscopic role of water in driving the protein properties was also investigated by means of FTIR spectroscopy, looking in particular at the O-H stretching band [60,84] which is mainly dominated by water contributions [64]. In Figure 6, we report the O–H stretching spectra of hydrated lysozyme with h = 0.3 at several temperatures in the range 180 K < T < 350 K. It is well known that the low-frequency contributions belong to structured water, that is water arranged in low-density local structures, whereas those in the high-frequency region belong to free molecules [64,85]. We also report in the figure the data of bulk water at 293 K (black line) in order to make a comparison. In particular, it is noticeable how the O–H stretching band is broader for bulk water with respect to hydration water confirming its limited configurational degrees of freedom. Furthermore, its central frequency is red-shifted by about 50 cm${}^{-1}$ with respect to that of bulk water confirming the existence of more disordered local structures in the bulk condition.

It is worth noticing that the spectral evolution for the hydration water on increasing the temperature shows the presence of a high-frequency shoulder progressively pronounced starting from about 310 K. However, the contributions at lower frequencies decrease in intensity confirming the transition from structured water at low temperature to free water at high temperature, corresponding to the conformational changes experienced by the protein. By performing a careful spectral deconvolution by means of Gaussian functions, the trend with temperature of the different contributions was evaluated [64]. Note that the central band of the O–H stretching spectra corresponding to hydration water moves toward the higher frequencies as the temperature increases (Figure 6). The analysis of its thermal evolution for hydrated lysozyme with h = 0.3 and 0.37 shows three linear behaviors separated by the characteristic temperatures of about 225 and 320 K [84]. This suggests that the structure and dynamics of the hydrogen bond network that water develops with lysozyme, manifest two important vibrational changes, at least in the population of vibrating species, just at these temperatures. Again, T${}_{L}=225$ K marks the low-temperature limit of the protein biological activity because it corresponds to the water dynamical crossover temperature above which anharmonic motions, that were frozen below it, begin. This activation, as already mentioned, is observed for many hydrated bio-systems with different experimental and theoretical approaches looking for examples in the mean-squared atomic displacement [35,70,86]. The slope change at about 320 K is instead related to the onset of the water tetrahedral structure that, beginning to be stable just below it, triggers the formation of new energetically favored geometrical configurations. Furthermore, for solid-like systems, the changes in the volume expansion coefficient are correlated with the changes in the frequencies of normal modes. For ice, a correlation between the oxygen–oxygen distance and the O–H stretching frequencies has been observed [87]. Similarly, in our case, we can assume that the variation of the O–H stretching frequencies of hydration water are associated with the structural changes induced by the modification of the corresponding force constant and reflected in the behavior of the thermal expansion coefficient. Therefore, there is a correlation between the observed changes at T* and the corresponding thermodynamic properties of thermal expansion coefficient already discussed. At the same time, the structural variations of lysozyme are associated with the water dynamical transition at T${}_{L}$.

### 2.4. The Lysozyme Moieties: FTIR Results

It is well known that infrared spectra are characterized by several bands corresponding to specific peptide vibrational modes called Amides that can be studied to assign the protein secondary structure and to follow its changes [88,89]. The region of interest falls within the interval 1300–1800 cm${}^{-1}$ to which the so-called Amide I, II, and III modes belong. The Amide I vibration is centered at about 1650 cm${}^{-1}$ and mainly corresponds to the C=O stretching vibrational mode of the different structures of the protein backbone. In fact, the Amide I band can be deconvoluted in its principal contributions (see Table 1) and its shape strongly depends on the protein side chain configuration. The Amide II mode is centered at about 1550 cm${}^{-1}$ and is essentially due to the out-of-phase combination between the N–H in plane bending and the C–N stretching. In addition, this band indeed contains relevant information on the protein secondary structure and can be used to study conformational changes. The Amide III band lies in the frequency interval 1200–1400 cm${}^{-1}$ and corresponds to the superposition of different modes such as the combination of in-phase N–H bending with C–N stretching, C–O in plane bending and C–C stretching. Hence, it is very difficult to extract precise information from that mode.

In detail, here we investigate the thermal evolution of the vibrational modes that fall within the spectral region from 1500 to 1800 cm${}^{-1}$, that is Amide I and II. The different contributions, including those of the bending vibrations of water molecules, belonging to this region are assigned and reported in Table 1 [88,90].

**Figure 7:**
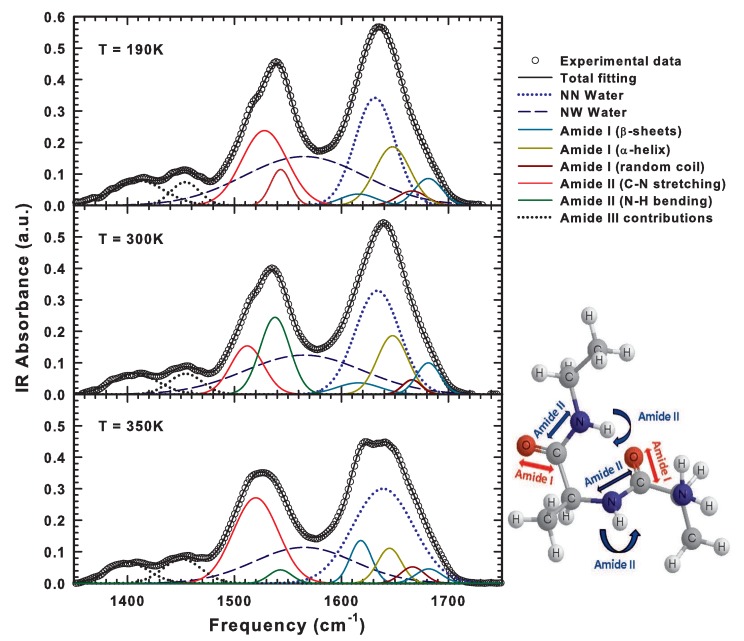
The Gaussian deconvolution of the recorded FTIR spectra for hydrated lysozyme with h = 0.3, at three different temperatures. We illustrate the case of T = 190 K (glassy state), T = 300 K (native state) and T = 350 K (irreversible denaturation). On the bottom right a schematic representation of the Amide I and II vibrational modes is shown. Figure adapted from Reference [67].

We have executed a deconvolution of the experimental bands of Amide I and II into their sub-bands by also considering the water O–H bending contributions. The spectral features corresponding to the O–H bending of water molecules centered at about 1640 cm${}^{-1}$ shows a shoulder centered at about 1560 cm${}^{-1}$ that can be attributed to hydrogen-bonded water molecules, that is to water molecules that are embedded in clusters. Generally, this last contribution is more intense at low temperature and can be attributed to the portion of low-density liquid water local structures [67,91]. The “resonance” condition between water O–H bending and the Amide I and II vibrational modes allows an efficient energy transfer between water and protein. In fact, bending vibrational modes usually involve intermolecular interactions and indeed the corresponding energy transfer. For hydrated protein, this coupling thus occurs between water and the different amino acids. In particular, a high efficiency was shown for precise amino acidic residues such as asparagine and glutamine whose presence was related to a progressive increase in protein structural instability [92]. In Figure 7, we report the obtained deconvolution for hydrated lysozyme with h = 0.3 at three chosen temperatures in the three different regimes identified by means of the analysis of the O–H stretching frequencies. We also include an over-simplified representation of the main vibrational contributions to Amide I and II modes. Note that the recorded spectra show interesting differences that are associated to the corresponding state of the system. In particular, at the lowest temperature, the system is in a glassy state: the bands corresponding to Amide I and II are more intense and narrower with respect to the higher temperatures. However, the same bands appear progressively broader and less intense on increasing the temperature within the whole considered range. In particular, above the denaturation threshold, at 350 K the Amide I band shows an intense shoulder centered at about 1620 cm${}^{-1}$ which is associated with the formation of “antiparallel” intermolecular β-sheets and to the onset of aggregation [91]. This result is in agreement with those obtained in a recent experimental investigation looking at the unfolding of lysozyme adsorbed to different lipid bilayers [93] in which the integrated peak area of the mentioned shoulder (from 1624 to 1604 cm${}^{-1}$) was used to follow the aggregation process.

**Figure 8:**
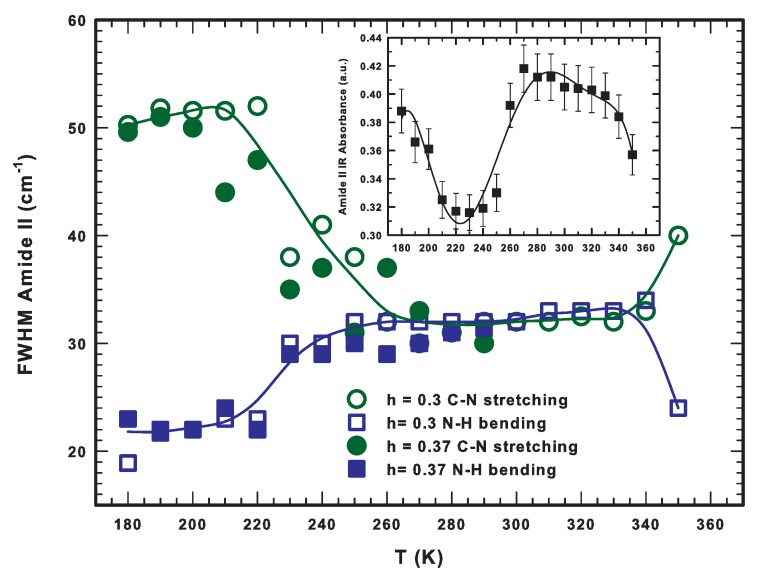
The Full Width at Half Maximum (FWHM) of the C–N stretching (1520 cm${}^{-1}$) and N–H bending (1540 cm${}^{-1}$) contribution of the Amide II band as a function of the temperature. The inset reports the total area of the whole band. Solid lines are a guide for the eye. Figure adapted from Reference [52,67].

Taking into account the previous evidence, we analyzed the behaviors of both the integrated areas and the full width at half maximum (FWHM) for all the Gaussian contributions obtained by an iterative fitting as a function of the temperature [67]. For what concerns the FWHM, we report in Figure 8 its behavior for the two contributions, that is N–H bending and C–N stretching at the two studied hydrations (h = 0.3 and 0.37). The inspection of the figure reveals the occurrence of a transition from two different regimes at T ≈ 225 K. Below this temperature, in fact, the FWHM of the C–N stretching mode is about 50 cm${}^{-1}$ whereas that of the N–H bending is narrower being about 20 cm${}^{-1}$. These values remain essentially constant until 220 K. Above it, the FWHM of the N–H bending increases sharply to the value of about 30 cm${}^{-1}$ which is also reached by the FWHM of the C–N stretching but in a smoother way covering about a window of 50 K. Then, for T > 270 K, the distribution of the two modes is approximately the same and the value of their FWHM slowly increases with the temperature up to 350 K, that is above the denaturation threshold, where the band of the C–N stretching mode becomes about two times larger than that of N–H bending. This means that the energy transfer between the bending modes of water O–H and peptide N–H is no longer favored because of the irreversible unfolding. This is accompanied by a sharp increase in the contribution related to the antiparallel β-sheets (see the appearance of the shoulder at about 1620 cm${}^{-1}$ shown in Figure 7 at 350 K) and a corresponding decrease to that of α-helices. As a consequence of this helix-to-sheet transformation the protein stability ceases and protein aggregation phenomena begin.

The protein stability, as well as the lifetime of hydrogen bonds, decreases on increasing the temperature. This provokes a sudden weakening of hydrophilic interactions reflected in the observed heat capacity behaviors [94]. The inset of Figure 8 reports the thermal behavior of the total spectral area of the Amide II band. This quantity shows a minimum at approximately 225 K, a maximum at about 270 K, and finally a sharp decrease above 340 K caused by the irreversible denaturation process. All the characteristic thermal thresholds mark important variations in the properties of the system reflected by the behavior of the considered quantities. The relative area of the two main modes of Amide II shows analogous behaviors of the corresponding FWHM reported in Figure 8 [67]. For T < 225 K, the C–N stretching dominates over the N–H bending mode and the relative area ratio is 6:1. For 225 < T < 273 K, the trend is the opposite with a ratio of approximately 1:2. When the protein denatures (T ≈ 350 K), this ratio is reversed again up to 15:1. This reinforces the idea that hydrogen atoms belonging to the peptidic N–H groups exchange with hydrogen atoms from the water molecules only in the unfolded state, whereas in the protein native state such an exchange is hindered especially for those amino acids that are buried in the protein core. It is worth noticing that, as it is well known, the most important hydrogen bonds that water establishes with proteins are those with the oxygen of carbonyl groups (C=O) and with the hydrogen of amide groups (N–H). In particular, in the first case, water acts as a proton donor whereas in the second case as a proton acceptor [95]. Hence, the same water molecule can be contemporary hydrogen bonded both with the carbonyl oxygen and with the hydrogen of the secondary amine group (N–H). In such a way, water can bridge these two molecular groups playing a fundamental role during protein folding, in protein–protein binding, and also in molecular recognition. As a matter of fact, the protein folding is accompanied by the formation of hydrophobic clusters whose presence can compensate the loss of configurational entropy for the system.

### 2.5. The Lysozyme Moieties: NMR Results

**Figure 9:**
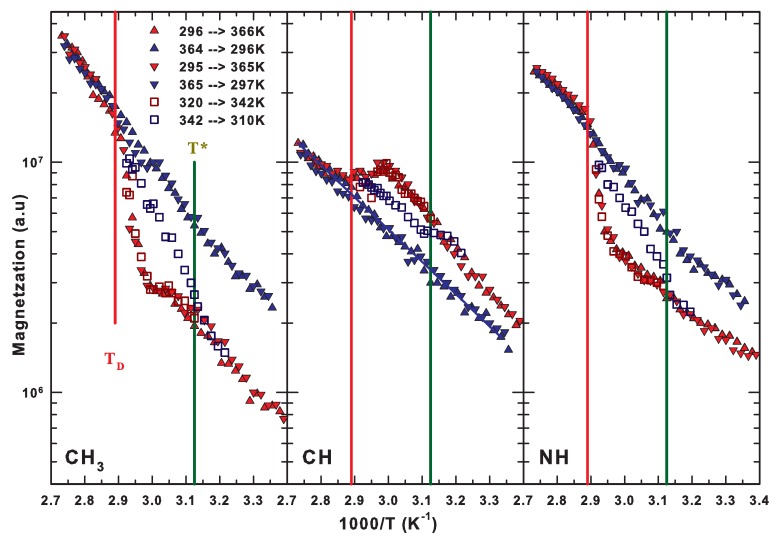
The behavior of the magnetization of the different considered molecular groups during different thermal cycles as a function of the inverse temperature in an Arrhenius fashion. The left panel reports the behavior of the highly hydrophobic methyl group, the central panel that of the methine group, and the right panel that of the hydrophilic secondary amine group. Figure adapted from Reference [11].

NMR spectroscopy, by taking advantage of the HR-MAS technique, allowed us to investigate the whole folding/unfolding process including its reversibility from the point of view of the protein functional groups by performing various measurements consisting of several thermal cycles. By choosing different starting and ending temperatures, we considered both hydrophobic and hydrophilic groups in order to follow their different pathways within the energetic landscape. As mentioned, we considered complete thermal cycles beginning in the native state at about 300 K and with a thermal inversion point above the denaturation threshold at about 365 K. For the investigation of the irreversibility region, we inverted the thermal cycle below T${}_{D}$. The enhanced resolution enabled us to discriminate the different contributions of protonated groups within the NMR signal. In particular, we focused on the magnetization of the signals centered at about 0.8 ppm (methyl group, CH${}_{3}$), 0.94 ppm (methine group, CH), and 6.7 ppm (secondary amide group, NH). Figure 9 shows in three different panels an Arrhenius plot of the magnetization of the considered molecular groups during different thermal cycles as a function of the inverse temperature. In all panels, the values of the relevant temperatures T${}_{D}\approx 346$ K and T* ≈320 K are highlighted by a red and a green line, respectively. We recall here that T* is the temperature at which the isothermal compressibility of water has its minimum value and does not depend on the pressure. T*, marking the breakdown of the tetrahedral structure of water, corresponds to the thermal limit of the protein native state. From Figure 9, one can see that the magnetization behavior for the CH${}_{3}$ and NH groups is quite similar, whereas that of the CH group is very different. In fact, in such a case, the values regarding the heating path are more intense than those of the cooling path at the same temperature, in the range T < T${}_{D}$. This indicates the existence of very different routes within the energetic landscape describing the folding/unfolding process of these groups. Note that the larger variation is shown by the magnetization of the methyl group that on increasing the temperature displays a first inflection just at T* (left panel). The sharp variation occurs, however, for all three groups at about 340 K and ends in a linear behavior for temperatures above T${}_{D}$≈ 346 K.

Within our approach, we evaluated the different activation energies for each functional group along all the pathways of the process (e.g., Native, Reversible, and Irreversible) [11]. In detail, in the case of the methyl group, we have found an activation energy of about 8.3 kcal/mol during the heating path (red symbols in Figure 9) below T* (solid green line). Then, within the reversible unfolding region, the magnetization at first remains nearly constant until about 340 K and then increases sharply with an activation energy of about 50 kcal/mol up to T${}_{D}$. Finally, for temperatures higher than T${}_{D}$, it increases with a corresponding activation energy of 8.69 kcal/mol that also remains the same during the cooling path (closed blue triangles). Instead, if the thermal cycle is inverted below T${}_{D}$, the magnetization follows a very different cooling path with an activation energy of about 12 kcal/mol, and below T* it recovers the values assumed during the heating path. This confirms the reversibility of the unfolding process of hydrated lysozyme for temperatures lower than about 346 K.

The middle panel of Figure 9 reports the thermal behavior of the magnetization for the methine group (CH). It increases on heating until about 340 K above which it slowly decreases up to T${}_{D}$ and then it increases again towards the temperature of inversion of the cycle. As already mentioned, it greatly differs from that of the two other considered groups because in the heating path it is more intense with respect to the cooling for T < T${}_{D}$. This indicates that the methine groups are more mobile in the heating phase of the native and reversible regions with respect to the irreversible unfolding state. Furthermore, this evidence suggests that the most hydrophobic groups (i.e., the methyl group) are buried within the protein core in its native state; at the same time the most hydrophilic groups (i.e., the secondary amine group) are strongly hydrogen bonded with the solvent molecules. The activation energies of the corresponding pathways for the methine group during the whole folding/unfolding process of hydrated lysozyme are 9.09 kcal/mol on heating the native state up to about 340 K, 6.41 kcal/mol when the protein is irreversibly unfolded (during the last part of heating and for all the cooling path), and about 5 kcal/mol for the cooling path in the case that the process can be reversible. For what concerns the N–H group (right panel of Figure 9), its magnetization increases monotonically until about 340 K with an activation energy of about 4.58 kcal/mol. Then, there is a sharp increase with the same rate of that of the methyl group up to 346 K, above which the magnetization evolves in an Arrhenius fashion with an activation energy of 7.38 kcal/mol that also persists during the cooling path but only until reaching T${}_{D}$. Below it, one can see two distinct Arrhenius behaviors separated by T*. This at higher temperatures has an activation energy of about 9.91 kcal/mol, whereas below T* is of about the same value of the heating path in the corresponding thermal region. Note that the energetic behavior shown by the partial thermal cycle, that reverses at approximately 342 K, i.e., 4 K below T${}_{D}$, has a completely different cooling path. It is characterized by an activation energy of about 12 kcal/mol and again at T* recovers the values of the native state (T < T*). It is noteworthy that the values we obtained for the energy and enthalpy difference between the native and unfolded states (50 and 12 kcal/mol, respectively) are in strong agreement with those calculated for lysozyme (58 and 14 kcal/mol) and for globular proteins [96].

We end this section by stressing that our NMR findings unambiguously indicate that T* and T${}_{D}$ respectively mark the low- and high-temperature limits of the reversible unfolding. Inside this thermal region, the energetic pathways within the energy landscape are compatible with a persistent folded structure imposed by the interplay between hydrophilic and hydrophobic interactions. For temperatures higher than T${}_{D}$, where the hydrogen bonding is no longer effective, the thermal disorder and van der Waals interactions favor a complete protein denaturation.

## 3. Materials and Methods

We studied hydrated hen egg white lysozyme with hydration level corresponding to the first hydration shell. In such a way we were able to focus on the most important interactions exerted between the protein and the water molecules. Samples were prepared by hydrating dried protein powders isopiestically at 5 ${}^{{}^{\circ}}$C in a closed chamber with 100% relative humidity until the hydration levels of h = 0.3 and 0.37 were achieved. In our experiments we used two different techniques that enabled us to obtain details underlying the folding/unfolding process of proteins. FTIR measurements were performed at atmospheric pressure using a PerkinElmer Spectrum GX Fourier transform spectrometer with an attenuated total reflection geometry. Spectra were recorded with a resolution of 4 cm${}^{-1}$ by performing 250 repetitive scans. The temperature scan was started at 180 K; we acquired the spectrum each 10 K up to 350 K by maintaining a stability of 0.1 K.

NMR experiments were performed by using a Bruker AVANCE 700-MHz NMR spectrometer. We acquired ${}^{1}$H NMR spectra of hydrated lysozyme with h = 0.3 as a function of temperature in diverse heating–cooling cycles with an accuracy of ±0.1 K controlled by using a cold nitrogen flow and a heating element. The complete investigated temperature range was 298–366 K, with 1 K steps for those thermal cycles that invert before T${}_{D}\approx$ 345 K and 2 K steps for the complete cycles that invert after the irreversible denaturation. The chemical shift of ethylene glycol was used for temperature calibration. Since the samples were essentially hydrated powders, in order to enhance the spectral resolution, we used the experimental technique known as High-Resolution Magic Angle Spinning. This is a very powerful NMR experimental technique to study semi-solid systems such as low-hydrated protein powders. It takes advantage of spinning the sample holder at high frequency (starting from 4000 rpm for biological materials) while it is tilted at about 54.74${}^{{}^{\circ}}$ with respect to the direction of the static magnetic field. This last condition ensures that the Hamiltonian terms, including the dipolar coupling which is responsible for broadening NMR peaks, can be neglected and the NMR signal is more resolved [97]. The possibility to study intact tissues or bio-systems without the need for any chemical treatment has aroused great interest towards this technique [98,99,100,101]. Finally in our experiments, hydrated protein powders were placed in a 50 μL rotor and spun at 4000 Hz to enhance the field homogeneity. We used 64 k points in the time domain, 128 scans and a minimum relaxation delay of 2 s. All spectra were processed (line broadening, Fourier transform, phase correction, and baseline adjustment) by using the standard routines of the Bruker software Xwinnmr version 3.5. Further details on sample and experimental methods can be found elsewhere [11,51,52,60,67,84].

## 4. Conclusions

In this work, we review our recent results obtained on hydrated lysozyme by means of FTIR and NMR experimental techniques. Our findings indicate that the hydrogen bonds formed between water molecules and both the carbonyl oxygen (C=O) and the secondary amide hydrogen (N–H) of protein or peptide trigger the low temperature (≈225 K) dynamic transition, below which the protein is not active. At 350 K, above the lysozyme denaturation threshold, the signal from antiparallel β-sheets is enhanced. The onset of this contribution marks the helix-to-sheet transformation and the subsequent processes associated with protein aggregation. The thermal behavior of the FWHM of the Amide II modes highlights the thermal regions where the energetic transfer with the O–H bending mode of water is favored, that is just when lysozyme is in its native state up to the reversible region of the unfolding process. Water molecules begin to loose their ability to develop stable hydrogen bonds for T > T* ≈ 320 K: their lifetime decreases on increasing the temperature.

Our NMR experiments shed light on the different energetic evolutions of both hydrophilic and hydrophobic moieties of hydrated lysozyme during the processes of folding and unfolding. The magnetization of the CH group shows contrasting behavior with respect to that of both the CH${}_{3}$ and NH groups, evidencing an enhanced mobility for the former. This is caused, on the one hand, by the burial effect that hydrogen bonding produces mainly on the more hydrophobic methyl groups, and on the other hand, by the stronger hydrogen bonds formed by water with the secondary amine groups.

Future studies aiming to rationalize the energetic behaviors of similar processes, such as those involving the formation of amyloid fibrils, may take advantage of these approaches and corresponding results. Furthermore, our findings may help understand the role of water in the protein function of organisms such as hyperthermophiles and psycrophiles living in extreme temperatures [102], as well as its mediation in protein-sugar systems [103,104].

## Figures and Tables

**Table 1:** Assignment of the principal IR bands in the spectral region of Amide I and II between 1500 and 1700 cm${}^{-1}$.

IR Frequency (cm${}^{-1}$)	Conformation	Assignment
1680–1696	Antiparallel β-sheets	C=O Stretching
1655–1675	Random coil	C=O Stretching
1650–1657	α-helix	C=O Stretching
≈1640	Free water molecules	O–H Bending
1612–1630	Antiparallel β-sheets	C=O Stretching
≈1560	Hydrogen-bonded water molecules	O–H Bending
≈1540	Peptide backbone	N–H Bending
≈1520	Peptide backbone	C–N Stretching

## Abbreviations

The following abbreviations are used in this manuscript:

FTIR	Fourier Transform Infrared Spectroscopy
NMR	Nuclear Magnetic Resonance
FWHM	Full Width at Half Maximum
